# Integrated Community-Based Health and Social Care Interventions for Older People: A Key Policy Priority for All Countries: Comment on "The Effect of Integrated Care After Discharge From Hospitals on Outcomes Among Korean Older Adults"

**DOI:** 10.34172/ijhpm.9057

**Published:** 2025-09-17

**Authors:** Peter Lloyd-Sherlock

**Affiliations:** School of Healthcare and Nursing Sciences, Northumbria University, Newcastle upon Tyne, UK.

**Keywords:** Older People, Health Services, Long-Term Care, Intersectoral

## Abstract

This paper situates Choi and Yoo’s study within broader sets of policy challenges related to rapid increases in numbers of older people in need of long-term care (LTC). These challenges include a need to fundamentally transform health services and to develop fully integrated systems of health and LTC for older people. The paper argues that Korea’s Integrated Pilot Project (IPP) is a key step in this direction, along with similar interventions in Thailand and Brazil. Choi and Yoo’s study adds to a wider body of evidence that these interventions enhance health system efficiency at the same time as improving the lives of older people and their family caregivers.

 Choi and Yoo’s study^1^ is highly relevant for researchers and policy-makers in any country with substantial or fast-growing numbers of older adults. This includes all high-income and middle-income countries, as well as many low-income ones.^2^

 Current approaches to meeting the needs of older adults continue to prioritize funding for hospital services instead of care provided in other settings. Since the Alma Ata Conference in 1978, it has been widely acknowledged that this approach is inefficient, unsustainable and inequitable.^3^ However, little progress has been made towards reorienting health systems, due to a variety of political and structural barriers.^4^ Rapid population ageing has added urgency to this healthcare reform agenda. In the UK, for example, numbers of hospital admissions increased by 49% between 1999 and 2019, with older people accounting for the majority.^5^ In a similar way, many countries have attempted to shift reliance on residential forms of long-term care (LTC) towards forms of support that enable older people to remain in their own homes. This has been driven by cost concerns and evidence about older people’s own preferences.^6^ More broadly, there is growing recognition of the need to understand health and LTC services for older adults as a single endeavour, requiring closer integration between health and social welfare agencies.^7^ Nevertheless, this form of intersectoral collaboration is often difficult to achieve in practice.^8^

 The government of Korea had already done much to address these challenges before its Integrated Pilot Project (IPP) began in 2019. Most notably, it had established a national LTC insurance scheme. This scheme had led to a rapid expansion of services, especially residential care, including large numbers of hospitals specialising in care for older people.^9^ As such, it reinforced existing inefficiencies in health and LTC provision. The IPP was therefore motivated by a desire to shift provision towards community-based services and enable older people to remain in their homes.

 Choi and Yoo’s study provides robust evidence for a range of highly relevant outcomes. Reducing emergency visits by older adults is associated with more efficient utilisation and allocation of health services. Studies from other countries report that community-dwelling older adults with comorbid conditions account for a high share of emergency presentations at hospitals and primary healthcare.^10,11^ Choi and Yoo also report older people in IPP spent on average 35.2 more days living at home during the 505.5-day study period. This outcome reflects reductions in hospital admissions, length of stay for each admission and the risk of being admitted to a residential LTC facility. In other words, IPP reduced pressures on hospital services and permitted older people to remain at home, where most preferred to be.

 The data on cost savings associated with these effects are especially compelling: An annual reduction of US$ 4835 per person. These savings should be offset against the costs of IPP itself, but estimates are not available, reflecting the complexity of costing an intervention involving customised components and multiple agencies. When considering value for money, it should be acknowledged that IPP’s benefits may go beyond reduced health and LTC expenditure to include quality of life enhancement for older people. As well as remaining in their own homes, the intervention may address loneliness and social isolation, which are increasingly prevalent among older people in Korea.^12^ IPP is also likely to benefit family caregivers. A large share of caregiving for older people in Korea is still provided by mainly female family members.^13^ Regular home care visits, part of the pilot intervention, may offer a degree of respite and support to this invisible army of caregivers.

 Choi and Yoo’s study provides valuable evidence to guide future policy in Korea once the second phase of the pilot concludes in 2025. It also offers insights for policy-makers across the globe who face an urgent need to identify evidence-based options. Korea’s IPP shares some features with interventions in high-income countries offering home-based healthcare to older people discharged from hospital.^14^ A key difference, however, is that IPP combines short-term post-discharge clinical services with more continual provision of social care, which continues so long as there is an assessed need. It is not possible to assess whether the main effects demonstrated by Choi and Yoo’s study can be specifically attributed to either the clinical or the social care elements of IPP. The likelihood is that they are holistic, reflecting synergies across the whole intervention package, but there is a need for more in-depth qualitative research to assess these effects.

 In many respects, IPP shares more similarities with schemes found in some middle-income countries. Thailand, for example, began to develop small pilots of similar interventions in 2003 which evolved over time into a community-based program of health and social care reaching over 130 000 older people.^15^ Curiously, the initial Thai pilots were developed with support from Korea’s international development agency, and it is possible that this experience later informed the development of IPP back home.

 In Brazil, *Programa Maior Cuidado* (PMC), was initially developed in one city, Belo Horizonte, and is now being extended with support from the Ministry of Health and local governments.^16^ Like IPP, Brazil’s PMC entails a close partnership between health and social welfare agencies, coordinated at local government level. Similarly, PMC has generated substantial cost savings and health service efficiencies such as shorter hospital stays and reduced emergency health service use.^17^ Evaluations of PMC also show substantial effects on family caregivers, including improved physical and mental health.^16^ Home visits by trained carers are often used by family caregivers as an opportunity to manage their own medical appointments and to learn strategies (such as lifting older people) which pose less risk to their own health. Just as for many older people, family caregivers dealing with round-the-clock care needs often remain trapped in the home and experience high levels of social isolation and stress. As such, the benefits of PMC for family caregivers are just as important as those for older people with care needs.

 The experiences of schemes such as these in Korea, Brazil, and Thailand provide two key insights. First, the practical challenges of developing community-based services combining health and social welfare agencies are often considerable. Secondly, that the potential benefits of persisting are huge, including reduced pressure on health services and improved quality of life for older people as well as their caregivers. Other countries need to urgently consider similar strategies.

## Ethical issues

 Not applicable.

## Conflicts of interest

 Author declares that he has no conflicts of interest.

## References

[1] Choi JW, Yoo AJ (2024). The effect of integrated care after discharge from hospitals on outcomes among Korean older adults. Int J Health Policy Manag.

[2] World Health Organization (WHO). Progress report on the United Nations Decade of Healthy Ageing, 2021-2023. Geneva: WHO; 2023. https://www.who.int/publications/i/item/9789240079694.

[3] Hone T, Macinko J, Millett C (2018). Revisiting Alma-Ata: what is the role of primary health care in achieving the Sustainable Development Goals?. Lancet.

[4] Chokshi DA, Cohen L (2018). Progress in primary care-from Alma-Ata to Astana. JAMA.

[5] Naser AY (2023). Hospitalisation profile in England and Wales, 1999 to 2019: an ecological study. BMJ Open.

[6] Maarse JA, Jeurissen PP (2016). The policy and politics of the 2015 long-term care reform in the Netherlands. Health Policy.

[7] Lloyd-Sherlock P, Billings J, Aredes JS (2020). Meeting the complex challenge of health and social care provision for rapidly-ageing populations: introducing the concept of “avoidable displacement from home. ” Cad Saude Publica.

[8] Reed S, Oung C, Davies J, et al. Integrating health and social care. A comparison of policy and progress across the four countries of the UK. Nuffield Trust Research Report 2021. https://www.nuffieldtrust.org.uk/sites/default/files/2021-12/integrated-care-web.pdf.

[9] Kim H, Lim W (2015). Long-term care insurance, informal care, and medical expenditures. Journal of Public Economics.

[10] Surate Solaligue DE, Hederman L, Martin CM (2014). What weekday? How acute? An analysis of reported planned and unplanned GP visits by older multi-morbid patients in the Patient Journey Record System database. J Eval Clin Pract.

[11] Andrade V, dos Santos K, Dantas J, Dantas D, Assis Neves Dantas, R. Cuidados de urgencia e emergencia ao idoso na assitencia pre-hospitalar: revisao de escopco. 2019. https://www.researchgate.net/publication/342338855_CUIDADOS_DE_URGENCIA_E_EMERGENCIA_AO_IDOSO_NA_ASSISTENCIA_PRE-HOSPITALAR_REVISAO_DE_ESCOPO.

[12] Lee G, Kim C (2024). Social isolation and mental well-being among Korean older adults: a focus on living arrangements. Front Public Health.

[13] Park E, Kim N, Ta Park VM, Rhee Y (2020). Caregiving for community-dwelling older persons in South Korea: current formal and informal care use and expectation. J Appl Gerontol.

[14] Mas M, Inzitari M, Sabaté S, Santaeugènia S, Miralles R (2017). Hospital-at-home Integrated Care Programme for the management of disabling health crises in older patients: comparison with bed-based Intermediate. Care Age Ageing.

[15] Glinskaya E, Walker T, Wanniarachchi T. Caring for Thailand’s Aging Population. Washington, DC: World Bank; 2021; https://openknowledge.worldbank.org/server/api/core/bitstreams/c72dd727-e785-5748-a33b-83782b8da182/content.

[16] Lloyd-Sherlock P, Fialho de Carvalho P, Giacomin K, Sempe L. Technical Report on Programa Maior Cuidado, Brazil. Washington, DC: Inter-American Development Bank; 2024. https://publications.iadb.org/en/programa-maior-cuidado-integrated-community-based-intervention-care-older-people.

[17] Lloyd-Sherlock P, Fialho de Carvalho P, Giacomin K, Sempé L (2023). Addressing pressures on health services in Belo Horizonte, Brazil through community-based care for poor older people: a qualitative study. Lancet Reg Health Am.

